# B cells and T cells abnormalities in patients with selective IgA deficiency

**DOI:** 10.1186/s13223-023-00775-6

**Published:** 2023-03-20

**Authors:** Yasser Bagheri, Tannaz Moeini Shad, Shideh Namazi, Farzaneh Tofighi Zavareh, Gholamreza Azizi, Fereshteh Salami, Somayeh Sadani, Ali Hosseini, Mohsen Saeidi, Salar Pashangzadeh, Samaneh Delavari, Babak Mirminachi, Nima Rezaei, Hassan Abolhassani, Asghar Aghamohammadi, Reza Yazdani

**Affiliations:** 1 Cancer Research Center, Golestan University of Medical Sciences, Gorgan, Iran; 2 Department of Immunology, School of Medicine, Golestan University of Medical Sciences, Gorgan, Iran; 3 Research Center for Immunodeficiencies, Pediatrics Center of Excellence, Children’s Medical Center Hospital, Tehran University of Medical Sciences, 62 Qarib St., Keshavarz Blvd., Tehran, 14194 Iran; 4 Primary Immunodeficiency Diseases Network (PIDNet), Universal Scientific Education and Research Network (USERN), Tehran, Iran; 5 Department of Immunology, School of Medicine, Iran University of Medical Sciences, Tehran, Iran; 6 Department of Immunology, School of Public Health, Tehran University of Medical Sciences, Tehran, Iran; 7 Non-Communicable Diseases Research Center, Alborz University of Medical Sciences, Karaj, Iran; 8 Clinical Research Development Unit (CRDU), Sayad Shirazi Hospital, Golestan University of Medical Sciences, Gorgan, Iran; 9 Stem Cell Research Center, Golestan University of Medical Sciences, Gorgan, Iran; 10 Division of Gastroenterology, Department of Medicine, Duke University, Durham, NC USA; 11 Department of Immunology, School of Medicine, Tehran University of Medical Science, Tehran, Iran; 12 Network of Immunity in Infection, Malignancy and Autoimmunity (NIIMA), Universal Scientific Education and Research Network (USERN), Tehran, Iran; 13 Division of Clinical Immunology, Department of Biosciences and Nutrition, Karolinska Institute, Stockholm, Sweden; 14 Department of Neurology, Thomas Jefferson University, Philadelphia, PA USA

**Keywords:** Inborn errors of immunity, Primary immunodeficiency, Selective IgA deficiency, B cell subsets, T cell subsets, Flow cytometry, Proliferation assay

## Abstract

**Background:**

Selective IgA deficiency (SIgAD) is the most prevalent inborn errors of immunity with almost unknown etiology. This study aimed to investigate the clinical diagnostic and prognostic values of lymphocyte subsets and function in symptomatic SIgAD patients.

**Methods:**

A total of 30 available SIgAD patients from the Iranian registry and 30 age-sex-matched healthy controls were included in the present study. We analyzed B and T cell peripheral subsets and T cell proliferation assay by flow cytometry in SIgAD patients with mild and severe clinical phenotypes.

**Results:**

Our results indicated a significant increase in naïve and transitional B cells and a strong decrease in marginal zone-like and switched memory B-cells in SIgAD patients. We found that naïve and central memory CD4^+^ T cell subsets, as well as Th1, Th2 and regulatory T cells, have significantly decreased. On the other hand, there was a significant reduction in central and effector memory CD8^+^ T cell subsets, whereas proportions of both (CD4^+^ and CD8^+^) terminally differentiated effector memory T cells (T_EMRA_) were significantly elevated in our patients. Although some T cell subsets in severe SIgAD were similar, a decrease in marginal-zone and switched memory B cells and an increase in CD21^low^ B cell of severe SIgAD patients were slightly prominent. Moreover, the proliferation activity of CD4^+^ T cells was strongly impaired in SIgAD patients with a severe phenotype.

**Conclusion:**

SIgAD patients have varied cellular and humoral deficiencies. Therefore, T cell and B cell assessment might help in better understanding the heterogeneous pathogenesis and prognosis estimation of the disease.

**Supplementary Information:**

The online version contains supplementary material available at 10.1186/s13223-023-00775-6.

## Introduction

Selective IgA deficiency (SIgAD) is the most prevalent primary immunodeficiency disorder (PID) or inborn errors of immunity (IEI), identified by serum concentration of IgA below 7 mg/dL and normal concentrations of IgG and IgM in patients over four years of age. The majority of SIgAD patients are asymptomatic, although some of them manifest different clinical manifestations, including gastrointestinal and respiratory tract infections, allergic diseases and autoimmune disorders. The disease progression to common variable immunodeficiency (CVID) in a selected group of SIgAD patients with IgG subclasses deficiency or autoimmune disorders has been reported [1].

No specific causes for the pathogenesis of SIgAD have not been reported yet. However, defects in the process of IgA class switch recombination (CSR), IgA production and secretion, as well as the long-term survival of IgA, switched memory B cells and plasma cells of SIgAD patients have been identified in unsolved cases [2]. Defects in these immunologic processes are associated with abnormalities in the lymphocytes of SIgAD patients. Hence, the assessment of the lymphocytes, especially B cell and T cell subsets, could be valuable and helpful. Several studies have demonstrated B cell and T cell abnormalities in some groups of SIgAD patients [3, 4]. Regarding B cell subsets, a decrease in the number of switched memory B cells, IgA plasma cells and transitional IL-10^+^ regulatory B cells of SIgAD patients has been reported [5–8]. On the other hand, defect in some T cell subsets of SIgAD cases have been reported that is linked to insufficient IgA-producing B cells [5, 8, 9].

Flow cytometric immunophenotyping can play an important role in the diagnosis, prognosis, classification and management of patients with SIgAD. Hence, for the first time, we aimed to investigate the main subpopulations of B and T lymphocytes along with an evaluation of T cell function, to clarify the correlation between immunological characteristics and clinical manifestations in symptomatic patients with SIgAD.

## Material and methods

### Patients

A total of 30 available symptomatic SIgAD patients (from the Iranian IEI registry [10, 11]) and 30 age-sex-matched healthy controls (HCs) were included in the present study. The HCs were confirmed after clinical and laboratory evaluations to have no immunodeficiency or any underlying disease. The patients had referred to the Children’s Medical Center (Pediatrics Center of Excellence affiliated with Tehran University of medical sciences, Tehran, Iran). All patients were diagnosed with SIgAD according to the European Society for Immunodeficiencies [12]. The study was approved by the Ethics Committee of Tehran University of Medical and written informed consent was obtained from all the individuals (IR.TUMS.VCR.REC.1396.2018). Demographic, clinical manifestations and immunological data of the patients were documented in a questionnaire form.

### Classification of patients

To compare demographic, clinical and immunological data, the patients were categorized into two groups severe and mild (based on clinical manifestations) as well as consanguine and non-consanguine groups (based on parental consanguinity). The minimum inclusion criteria for a patient to be considered symptomatic were ten warning signs of the Jeffrey Modell Foundation. The patients were divided into two groups of mild and severe, as patients with severe infections (e.g., bloodstream, central nervous system, and deep-seated infections like osteomyelitis and arthritis), autoimmunity, or malignancy were categorized in the severe group and other complications were considered as a mild group. Since pneumonia and other respiratory infections are common in SIgAD patients and we wanted to check it as a parameter between severe and mild groups, so we did not categorize it in the severe group.

### Lymphocyte subsets assay

Peripheral blood mononuclear cells (PBMCs) were separated from 8 ml blood samples collected in sodium heparin tubes using a Ficoll-Hypaque density gradient centrifugation at 600 g for 25 min at 22 °C. For extracellular staining, PBMCs were split into 5-panel fractions and stained for 20 min at 2–8 °C in a dark place with each cocktail containing the monoclonal antibodies at optimal concentrations. The B1 panel was utilized to determine naïve (CD19^+^CD27^−^IgM^+^IgD^+^), IgM only memory (CD19^+^CD27^+^ IgM^++^IgD^−^), switched memory (CD19^+^CD27^+^IgM^−^IgD^−^) and marginal zone-like B cells (CD19^+^CD27^+^IgM^++^IgD^+^). The B2 panel was used to identify CD21^low^ B cells (CD19^+^CD21^−/low^CD38^−/low^IgM^+++^), plasmablast (CD19^+^CD21^−/low^CD38^++/+++^IgM^−^) and transitional B cells (CD19^+^CD21^+^CD38^++^IgM^+^). The T1 panel was used to classify naïve (CD4^+^ or CD8^+^ and CD45RA^+^CCR7^+^), effector memory (CD4^+^ or CD8^+^ and CD45RA^−^CCR7^−^), central memory (CD4^+^ or CD8^+^and CD45RA^−^CCR7^+^) and T_EMRA_ (terminally differentiated effector memory) T cells (CD4^+^ or CD8^+^ and CD45RA^+^CCR7^−^). The T2 panel was used to classify regulatory T cells (TregsCD4^+^CD25^+^FOXP3^+^CD127^−/low^). For intracellular staining, following the surface molecule staining, they were fixed and permeabilized throughout FOXP3/Permeabilization buffer (eBioscience, US) according to the manufacturer’s instructions for the following: Anti-Human FOXP3 (PE), Anti-Human IL-17 (PE), Anti-Human IL-4 (APC) and Anti-Human IFN-γ (FITC). All antibodies and isotype controls were purchased from eBioscience, US corporation.

To assess T helper cells (including Th1, Th2 and Th17), 1 × 10^6^ (cell/mL) of PBMCs were cultured within in the Roswell Park Memorial Institute (RPMI 1640) cell culture medium, followed by stimulation with phorbol myristate acetate (PMA, 50 ng/mL, Sigma-Aldrich, US) / ionomycin (1 μg/mL, Sigma-Aldrich), and in the presence of brefeldin (5 μg/mL, eBioscience). Then, the cells were incubated at 37 °C in 5% CO2 and 95% humidity incubator for 5 h. The stimulated cells were washed with phosphate-buffered saline (PBS), and surface staining with anti-human CD4 (PerCp)-Cy5.5 was performed. Intracellular cytokine staining antibodies (anti-IFN-γ FITC, anti-IL-17 PE and anti-IL-4 APC for evaluation of Th subsets and anti-FoxP3 PE for evaluation of Treg) were added and incubated at room temperature for 30 min. Cells were washed with permeabilization buffer, resuspended in cold staining buffer, and counts were determined using a BD FACSCalibur Flow Cytometer (BD Biosciences) (Additional file 1: Table S1). The gating strategy is similar to our previous study [13, 14].

### T cell proliferation assay

To assess T cell proliferation, PBMCs were cultured with fluorescent 5,6-carboxyfluorescein succinimidyl ester (CFSE, Biolegend, US). A CFSE stock solution was prepared by dissolving CFSE in dimethyl sulfoxide (DMSO) at a concentration of 5 mM/L, based on the manufacturer’s instructions. This stock was frozen in small aliquots to prevent excessive freeze and thaw cycles. CFSE was added to a transverse falcon tube containing 500μL of PBMC suspension (5 × 10^6^ cell/mL) in RPMI 1640 cell culture medium containing 10% FBS (fetal bovine serum, Biosera, France) with a final concentration of 5 μM and 2 mM L-glutamine, 100 U/mL penicillin and 100 μg/mL streptomycin (Lymphosep; Biosera, France). The tube turned rapidly and vortexed to ensure homogenous dispersal. After labeling, the cell suspension was incubated for 5 min at 37 °C. Then, 9 mL of RPMI1640 containing 10% FBS was added to the cell suspension and it was centrifuged at 500 g for 5 min. Cells were washed three times and 1 mL of RPMI1640 with 10% FBS was added. Regarding specific T cell stimulation and proliferation, anti-CD3 antibody (1 µg/ml) was added to 500 μL of sterile PBS, and the plate was incubated at 37˚C for 2 h. The coated plate was washed twice with sterile PBS and the labeled cells were directly added, and finally, anti-CD28 antibody (2 µg/ml) was added as a stimulant for T cells. The plate was incubated at 37 ºC in 5% CO_2_ and 95% humidity incubator for 96 h [15–17]. An unstimulated well was considered as a control for non-proliferative cells. After 96 h, the cells were harvested and washed. After staining with Anti-Human CD4 (PerCPCy5.5), the cells were eventually analyzed by BD FACSCalibur™ Flow cytometer and CellQuest Pro software (BD, Biosciences, San Jose, CA, US). Proliferation analysis was performed by comparing three criteria, including the percentage of cells divided (%divided), the average number of cell divisions that a cell undergoes (Division Index), and the average number of cell divisions that occur in the entire primary cell population (Proliferation Index) by FlowJo 7.6 software.

### Statistical analysis

SPSS software (Windows version 16.0; SPSS Inc., Chicago, IL, US) was utilized for statistical analysis. We used the Kolmogorov–Smirnov test to estimate whether data were normally distributed. The findings were presented as median (interquartile range [IQR], presented as a range with 25th–75th percentiles) values. A chi-squared test or Fisher’s exact test was utilized for comparisons. For comparison of more than two groups, if the data distribution was normal, ANOVA was used, and if the data distribution was non-normal, Kruskal Wallis’s test was utilized. The differences were considered statistically significant for *P*-values that were < 0.05.

## Results

### Demographic, immunologic and clinical findings

**Fig. 1:**
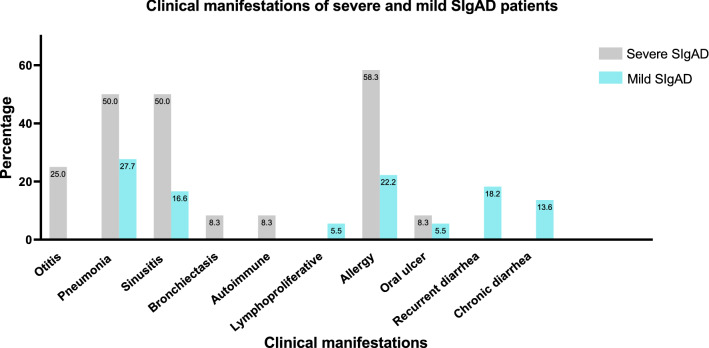
Comparison of clinical manifestations among severe and mild SIgAD

Among all registered Iranian SIgAD patients, 30 available patients (23 males and seven females) were recruited in the present study. The median (IQR) age of patients was 11 (7.3–16) years at the time of the study. Parental consanguinity was present in 16 (53.3% of) patients. Pneumonia (42.3%) was the most frequent clinical manifestation in the studied patients and malignancy was not diagnosed in this cohort. The demographic, clinical and immunologic characteristics of patients are summarized in Table 1. After categorizing patients based on severe and mild phenotypes, demographic, clinical and immunologic data were compared (Table 1, Fig. 1). The patients with severe phenotype manifested more respiratory complications than the mild group. Other parameters have not demonstrated a significant difference. The same comparisons were performed in patients with and without consanguinity, and none of them had a significant difference. In contrast, patients with mild phenotype presented a slightly higher rate of allergy and gastrointestinal manifestations with an increased serum IgE level, suggesting an increased switching toward IgE production.

**Table 1:** Comparison of demographic, clinical and immunological data of SIgAD patients with severe and mild phenotypes

Parameter	All patients (n = 30)	Severe (n = 12)	Mild (n = 18)	*p*-value
Age, year (IQR)	11 (7.3–16)	14 (6.3–34.5)	10 (7.7–14.2)	0.27
Age of onset, year (IQR)	4 (2–8.75)	8 (3–26)	3 (1.1–5.5)	0.05
Age of diagnosis, year (IQR)	8 (5.5–13.7)	9 (8–26)	8 (3.5–9)	0.06
Diagnostic delay, year (IQR)	2 (1.1–4.7)	3 (1–5)	0.9 (2–4.7)	0.8
Sex (Male/Female)	23/7	9/3	14/4	1.0
Lymphocytes, cell/ul (IQR)	4495 (3175–6325)	3650 (2300–5550)	4650 (4100–6725)	0.059
Lymphocytes % of leukocytes (IQR)	62 (43–77)	43 (39–58)	70 (55–78)	0.61
Neutrophil % of leukocytes (IQR)	45(37–56.5)	52.7 (46–63.7)	42.5 (32–51.5)	0.04
CD3% of lymphocytes (IQR)	60 (53–67)	53 (50.2- 63)	65 (57–70)	0.08
CD4% of lymphocytes (IQR)	35.5 (31–40.7)	33.5 (31.5–40.7)	36 (34–40.5)	0.38
CD8% of lymphocytes (IQR)	25 (19.7–30.5)	17.5 (16.2–20.2)	26 (22–31)	0.02
CD19% of lymphocytes (IQR)	16 (11.5–29.5)	15.5 (8.2–32.5)	16 (13.5–23)	0.81
IgG, mg/dl (IQR)	1380 (1006–1680)	1630 (1180–1931)	1216 (793–1534)	0.22
IgA, mg/dl (IQR)	0.5 (0–4.5)	2 (0–5.5)	0.3 (0–4.0)	0.77
IgM, mg/dl (IQR)	62.5 (59–119)	80 (118–175)	59.5 (49–92)	0.03
IgE, IU/ml (IQR)	43 (12.7–87)	32.5 (2.5–180)	49.5 (16.5–75)	0.84

IQR: Range with 25th percentile and 75th percentile; N: number. P < 0.05 were considered significant. Since the ages of the two groups of patients at diagnosis and at the time of the study were not significantly different, age was not a confounding factor in comparing clinical symptoms

### B-cell subsets

**Fig. 2:**
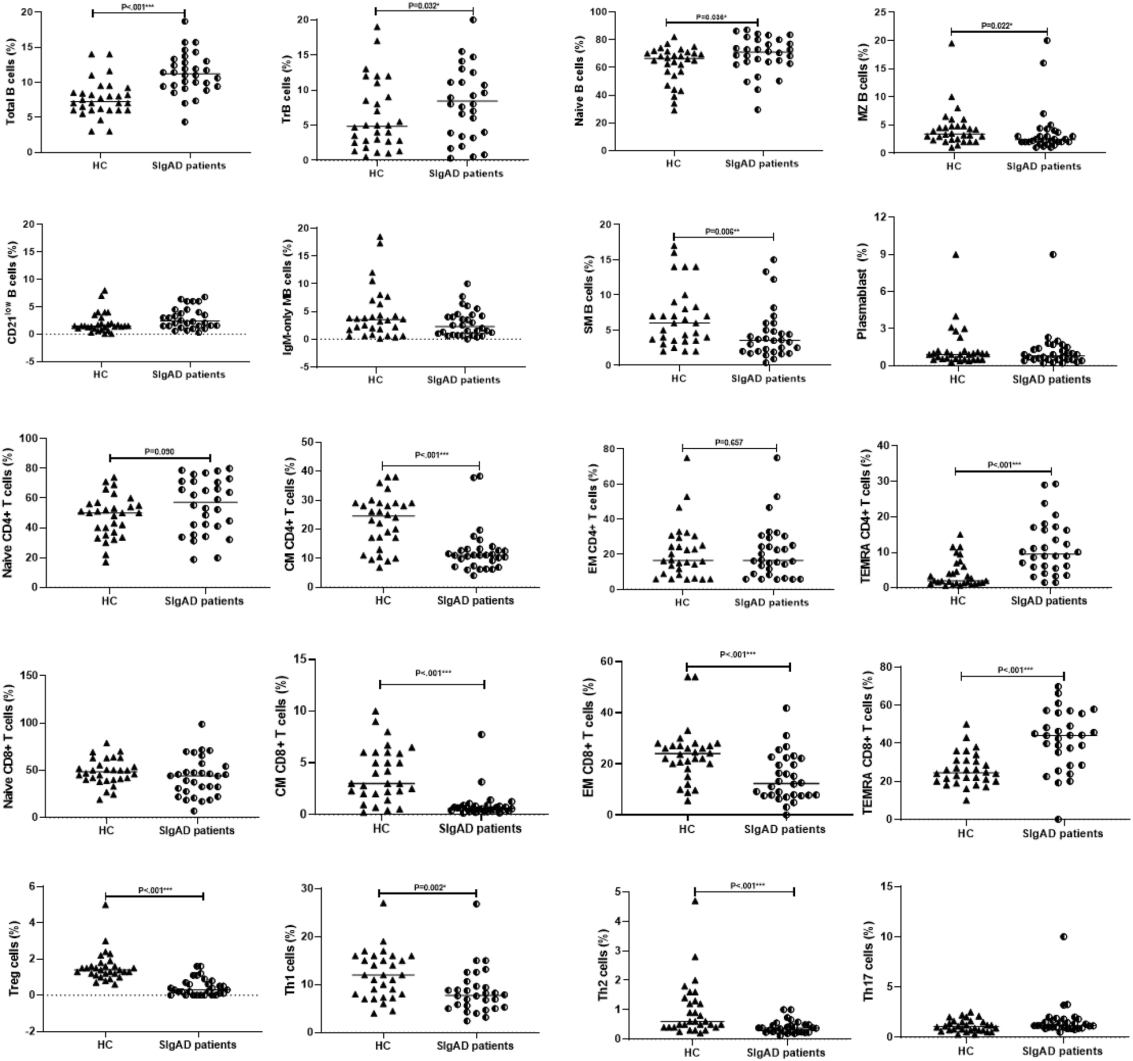
Quantitative analysis of B cell and T cell subset percentages in SIgAD patients and Healthy controls. The median is represented by a horizontal line. Data were analyzed using the Mann–Whitney *U* test. *p < 0.05, statistical significance between patients and HCs. *HC* Healthy control

**Fig. 3:**
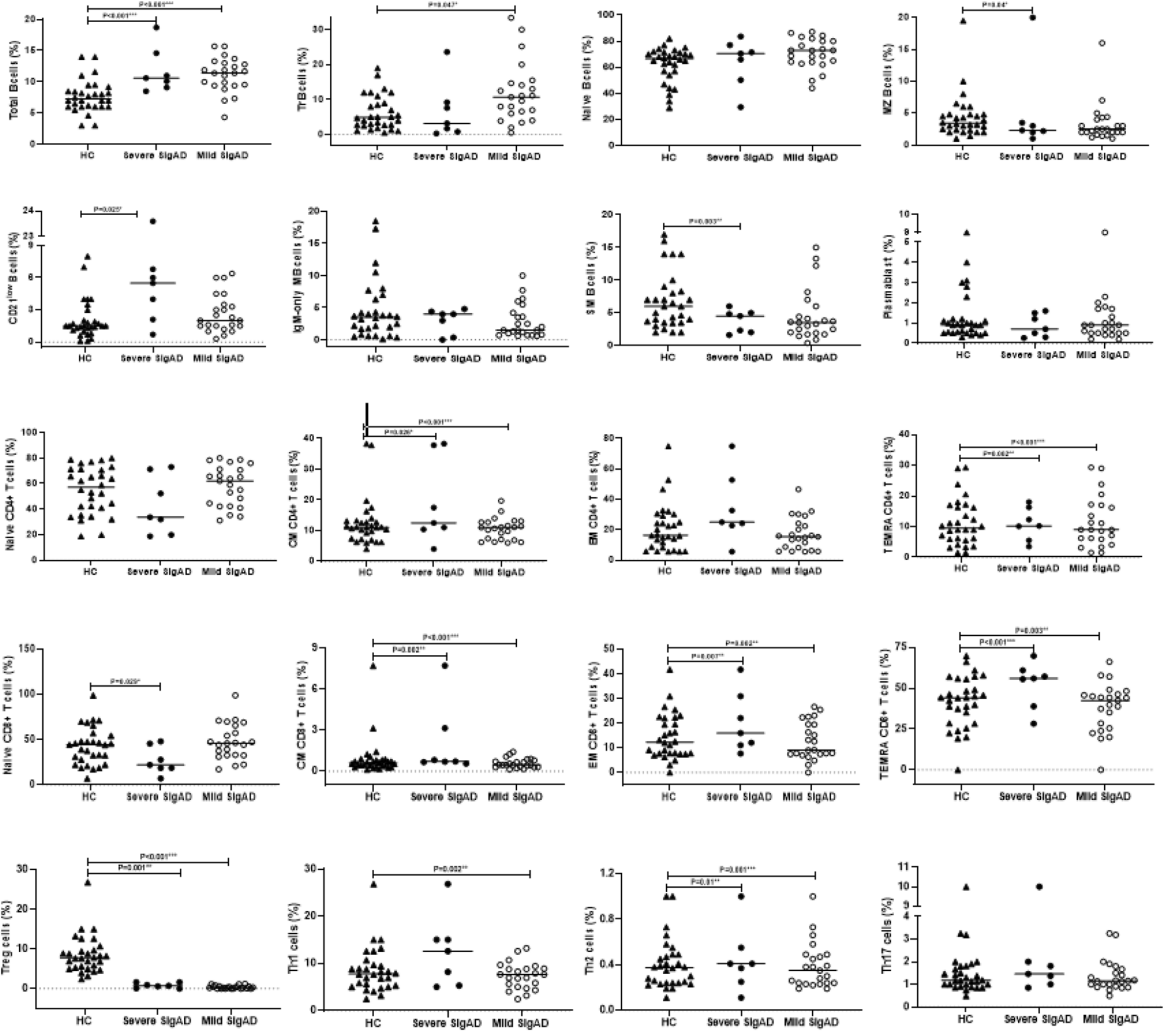
Quantitative analysis of B cell and T cell subset percentages in severe and mild SIgAD patients. The median is represented by a horizontal line. Data were analyzed using the Mann–Whitney *U* test. *p < 0.05, statistical significance between severe and mild patients

SIgAD patients showed significantly an increased frequency of CD19^+^ B-cells [11.2% (9.4–13.07%) vs. 7.2% (6–8.6%), *p* < 0.001], with increased naïve B-cells [71% (63.7–80%) vs*.* 66.5% (56.2–71.1%), *p* = 0.036] and transitional B-cells [8% (3.6–13.5%) vs. 4.8% (2.6–9.5%), *p* = 0.032] compared with HCs. In contrast, the percentage of marginal zone-like [2.3% (2–3.5%) vs. 3.4% (2.3–4.8%), *p* = 0.022) and switched memory B-cells [3.5% (1.9–5.5%) vs. 6% (3.5–8.4%), *p* = 0.006] were significantly lower than HCs. However, decreased IgM-only memory and plasmablasts and increased CD21^low^ B-cells in patients than those in HCs were not significant **(**Fig. 2). Interestingly, some comparisons were significant between patients’ clinical groups. The percentage of CD19^+^ B cells in both mild and severe phenotypes was significantly higher than HCs [11.6% (8.7–13) vs. 7.2% (6–8.6), *p* < 0.0001, 10.8% (9.6–13.2) vs. 7.2% (6–8.6), *p* < 0.0001], respectively. Severe SIgAD patients demonstrated a significant increase in the percentage of CD21^low^ B-cells [1.5% (1–2.2) vs. 2.7% (1.6–5.6),* p* = 0.025], and a significant decrease in the percentage of both marginal-zone and switched memory B cell subsets [3.4% (2.3–4.8) vs. 2.2% [2, 3], *p* = 0.040, 6% (3.5–8.4) vs. 2.7% (1.7–4.7), *p* = 0.003], respectively. The percentage of transitional B cells in mild SIgAD patients was higher than HCs [10.7% (3.9–13.8) vs. 4.8% (2.6–9.5), *p* = 0.047] (Fig. 3). The percentage of IgM-only memory was significantly higher in patients with consanguinity than in those without consanguinity. We also categorized the frequency of B cell subsets of SIgAD patients into three categories: normal, decreased and increased based on the normal range of HCs (Table 2). Based on this analysis, the most decrease in B cell subsets is related to switched memory B-cells (23%), while the most increase is related to naïve B cells (27%) in SIgAD patients. Except for increased CD21^low^ B-cells in severe SIgAD patients compared with mild SIgAD patients.

**Table 2:** Distribution of normal, increased and decreased proportions of T cell and B cell subsets in all SIgAD patients. N = 30

B cell subsets	Normal N (%)	Increased N (%)	Decreased N (%)
CD19^+^ B cells	26 (87%)	4 (13%)	0
Naïve B cells (CD19^+^, CD27^−^, IgM^+^, IgD^+^)	21 (70%)	8 (27%)	1 (3%)
Marginal zone-like B cells (CD19^+^, CD27^+^, IgM^++^, IgD^+^)	26 (87%)	2 (7%)	2 (7%)
Switched memory B cells (CD19^+^, CD27^+^, IgM^−^, IgD^−^)	23 (77%)	0	7 (23%)
IgM-only memory B cells (CD19^+^, CD27^+^, IgM^++^, IgD^−^)	29 (97%)	0	1 (3%)
CD21^low^ B cells (CD19^+^, CD21^−/low^, CD38^−/low^, IgM^+++^)	29 (97%)	1 (3%)	0
Transitional B cells (CD19^+^, CD21^+^, CD38^++^, IgM^+^)	24 (80%)	4 (13%)	2 (7%)
Plasmablasts (CD19^+^, CD21^−/low^, CD38^++/+++^, IgM^−^)	26 (87%)	1 (3%)	3 (10%)
CD4^+^ T cells	25 (83%)	3 (10%)	2 (7%)
Naïve T cells (CD4^+^, CD45RA^+^, CCR7^+^)	23 (77%)	6 (20%)	1 (3%)
Central memory cells (CD4^+^, CD45RA^−^, CCR7^+^)	21 (70%)	1 (3%)	8 (27%)
Effector memory cells (CD4^+^, CD45RA^−^, CCR7^−^)	22 (73%)	0	8 (27%)
T_EMRA_ cells (CD4^+^, CD45RA^+^, CCR7^−^)	20 (67%)	10 (33%)	0
Th1 (CD4^+^, IFN-γ^+^)	26 (87%)	1 (3%)	3 (10%)
Th2 (CD4^+^, IL-4^+^)	27 (90%)	0	3 (10%)
Th17 (CD4^+^, IL-17A^+^)	27 (90%)	3 (10%)	0
Regulatory T cells (CD4^+^, CD25^+^, FoxP3^+^, CD127^− /low^)	10 (33%)	0	20 (67%)
CD8^+^ T cells	28 (93%)	2 (7%)	0
Naïve T cells (CD8^+^, CD45RA^+^, CCR7^+^)	26 (87%)	1 (3%)	3 (10%)
Central memory T cells (CD8^+^, CD45RA^−^, CCR7^+^)	27 (90%)	0	3 (10%)
Effector memory T cells (CD8^+^, CD45RA^−^, CCR7^−^)	25 (83%)	0	5 (17%)
T_EMRA_ T cells (CD8^+^, CD45RA^+^, CCR7^−^)	18 (60%)	11 (37%)	1 (3%)

### T-cell subsets

The subset separation of CD4^+^ T cells revealed a significant reduction in total CD4^+^ T cells [36.5% (30.8–41.2%) vs. 40.1% (37.3–47.2%), *p* = 0.038)], central memory cells [11% (7.3–13.1%) vs. 24.5% (16–29%), *p* < 0.0001), Th1 [7.7% (5.2–9.9%) vs. 12% (7.8–16%), *p* = 0.002], Th2 [0.3% (0.2–0.4%) vs. 0.6% (0.4–1.3%), *p* < 0.0001] and Tregs [0.3% (0.03–0.7%) vs. 1.4% (1.1–1.6%), *p* < 0.0001] in patients compared with HCs. Oppositely, the percentage of T_EMRA_ [9.5% (5.7–16.4%) vs. 2% (1.3–6.2%), *p* < 0.0001] was meaningfully higher than HCs. Moreover, decreased effector memory and Th17, and increased naïve helper T cells in patients in comparison with HCs were not significant (Fig. 2). Regarding the percentage of CD8^+^ T cell subsets, central memory [0.6% (0.3–0.8%) vs. 3% (2–6%), *p* < 0.0001] and effector memory [12.3% (7.6–22.1%) vs. 23.9% (19.5–27.2%), *p* < 0.0001] were markedly diminished in patients compared with HCs. On the other hand, the percentage of cytotoxic T_EMRA_ [44.1% (28.4–55.7%) vs. 24.4% (20–31%), *p* < 0.0001] was significantly higher than HCs. However, increased total CD8^+^ T cells and decreased naïve CD8^+^ T cells were not significant in patients compared to those in HCs (Fig. 2).

Regarding the comparison of the percentage of CD8^+^ T cells subsets between severe SIgAD patients with HCs, naïve T cells, effector and central memory cells demonstrated a significant reduction. Also, there was a significant decrease in the percentage of total CD4^+^ T-cells, central memory, Th1, Th2, and regulatory T cells, whereas T_EMRA_ in both CD4^+^ and CD8^+^ T cells demonstrated an increase. On the other hand, the percentage of effector and central memory cells within CD8^+^ T cells, as well as central memory, T_EMRA_, Th1 and regulatory T cells within CD4^+^ T cell subsets demonstrated a significant decrease in mild forms of SIgAD compared to HCs. In contrast, we found an increase in T_EMRA_ CD8^+^ T cells and Th2 CD4^+^ T cells in mild patients compared to controls (Fig. 3). Comparisons of the percentages of all T cell subsets between SIgAD patients with and without consanguinity have not indicated any significant difference. We also categorized the frequency of T cell subsets of SIgAD patients into three categories: normal, decreased and increased based on a normal range of HCs (Table 2). Based on this analysis, the most decrease in T cell subsets is related to Tregs (67%), while the most increase is related to CD8^+^ T_EMRA_ (37%) in SIgAD patients. Flow cytometry results of B cell and T cell subsets in 30 SIgAD patients have shown separately in Additional file 1: Tables S2 and S3. To evaluate the impact of the T cell subset on the switching process of B cells and the production of different Ig subtypes we performed a correlation analysis. Surprisingly only IgG but not IgM and IgE were significantly associated with specific T cell subsets (negative association with naïve CD4 and naïve CD8 T cells and positive association with CD4^+^ T_EM_ and Th17 cells, Additional file 1: Table S4) indicating the independent association of IgM to IgE from co-stimulation of T cell subsets in our patient cohorts. Moreover, correlation analysis of absolute counts and percentage of subsets should significant direct correlation in all measured parameters (Additional file 1: Table S5).

### T cell proliferation

**Fig. 4:**
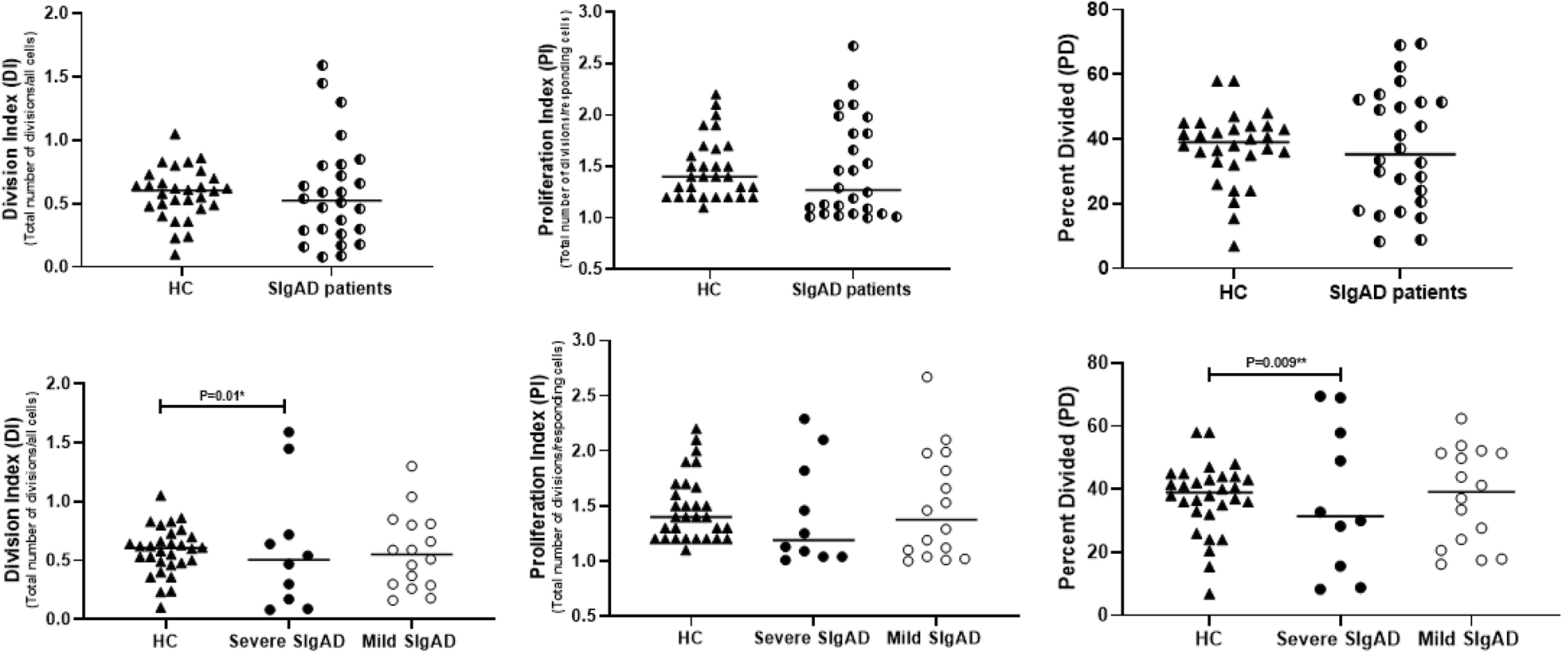
Comparison of T lymphocyte proliferation indexes in severe and mild SIgAD patients. The median is represented by a horizontal line. *p < 0.05, statistical significance between severe and mild patients

The data generated by CFSE labeled cultures was analyzed to quantify CD4^+^ T cell proliferation. There was no significant difference in division index (DI), proliferation index (PI) and percent divided (PD) between SIgAD patients and HCs (Fig. 4). Interestingly, when we compared these indexes between SIgAD patients with severe and mild phenotypes, we found that the median DI and PD in severe SIgAD patients in comparison with mild cases were significantly abrogated [0.1 (0.08–0.4) vs. 0.5 (0.3–0.8), *p* = 0.019 and 12.2 (8.4–26.4) vs. 42.5 (26.8–52.6), *p* = 0.009, respectively]. However, there was no significant difference in PI between severe and mild groups (Fig. 4). On the other hand, comparisons of DI, PI and PD between SIgAD patients with and without consanguinity were not significant.

## Discussion

SIgAD is the most prevalent IEI with various clinical manifestations. These patients have a different spectrum of clinical manifestations. Accordingly, immunologic investigations in patients with a different spectrum of clinical manifestations are helpful. The most prevalent clinical manifestation in IEIs, especially in SIgAD, is recurrent respiratory infections [18–20]. We found pneumonia as the most frequent complication in our registered symptomatic patients. Recurrent respiratory infections commonly manifest in the form of upper respiratory tract infection and may remain undiagnosed for several years; however, some SIgAD patients manifest more severe phenotypes such as bronchiectasis or obliterate bronchiolitis which force immunological investigation in these patients [21]. Given that recurrent respiratory infections have been reported as the most important cause of morbidity and death in children with IEIs, especially primary antibody deficiencies [22, 23], early diagnosis and management of respiratory disorders associated with SIgAD is very important [24, 25].

It has been indicated that abnormalities in B cell subsets are observed in some SIgAD patients [3, 4]. Our results indicated a significant increase in naïve and transitional B cells and a strong decrease in marginal zone-like and switched memory B-cells. This abnormal B cell pattern suggests defects in the terminal stages of B-cells differentiation, similar to CVID patients [26]. Given that CVID and SIgAD share almost similar genetic backgrounds and may accumulate as multiple cases within a family, this resemblance is predictable. In general, more than half of IEI patients registered in the Iranian registry have parental consanguinity, but this rate still is less common in SIgAD patients. In SIgAD Iranian patients compared to Western patients, consanguinity is more prevalent. Although monogenetic causes may exist, it has not been identified yet despite next-generation sequencing in several patients [27].

We detected a reduction in marginal zone-like and switched memory B-cells, especially in severe SIgAD patients, as has been previously reported [28]. Recently we reported similar reduction in marginal zone-like and switched memory B-cells in CVID patients [14]**.** On the other although we observed a significant decrease in switched memory in Ataxia Telangiectasia (AT) patients but a sharp increase in the marginal zone-like B cells was observed [29]**.** SIgAD patients, especially a group of patients with severe clinical manifestations (recurrent and intensive infection, and autoimmunity), have lower switched memory B-cells [28, 30, 31]. It has been suggested that the decrease in switched memory B-cell subpopulation is due to defects in the level of antibody class-switching recombination (CSR) process, caused by enzymatic deficiency, or abnormalities in the cytokine networks and their receptors [28]. Some SIgAD patients with severe phenotype progress to CVID, which reflects this subgroup of SIgAD may share with CVID common immune pathogenesis, particularly in the development of CSR step. Accordingly, switched memory B-cells are considered a diagnostic biomarker in patients [28]. However, the frequency of switched memory B-cells is normal among children in our study population, and the reduction was observed more in adult patients; suggesting that aging probably leads to the progression of SIgAD to CVID, especially in patients with severe clinical manifestations (data not shown). On the other hand, marginal zone B cells are a specialized population of B cells that produce IgM for the protection against infections, especially encapsulated bacteria [32]. Although previous studies have shown that the number of marginal zone-like B cells in SIgAD patients was not different compared to normal controls [33], nevertheless, we obtained a significant reduction in marginal zone-like B cells in our cases, similar to a previous report in CVID patients [34]. Reducing marginal B cell subsets in other patients with antibody production defects could be associated with an increased risk of infection such as pneumonia and a decrease in serum IgM levels, similar to CVID patients [35].

We found increased CD21^low^ B cells compared to control, mainly in severe SIgAD patients. Previous studies have reported an increase in CD21^low^ B cells in both SIgAD [3] and CVID patients [36], and other autoimmune diseases [37]. Recently also has been reported increase in the CD21^low^ in AT patients [29]. An increase in the number of CD21^low^ cells is directly related not only to autoimmunity but also to infection [36]. On the other hand, chronic exposure to viral infection may lead to the conversion of antigen-reactive B cells to unresponsiveness CD21^low^ B cells [38]. To clarify the cause of expanded CD21^low^ B cells; it is necessary to make further investigations for this B cell subpopulation. Given the high subpopulation of CD21^low^ B cells in CVID patients and the progression of some patients with SIgAD to CVID, the severe group of SIgAD patients with increased CD21^low^ B cells will more likely develop CVID. Therefore, they need a more regular follow-up to assess the course of the disease.

Transitional B cells are at an intermediate stage in the development between bone marrow immature cells and mature B cells in the spleen [39]. In the present study, we observed significantly increased transitional B cells in our SIgAD patients, especially in severe SIgAD patients, although the number of transitional B cells in children with SIgAD was normal (data not shown). We recently observed significantly decreased of the transitional B cells AT [29]. In contrast to previous studies that showed decreased transitional B cells [8, 28, 40], adult patients indicated slightly increased transitional B cells. Moreover, Lemarquis et al. showed a decrease in the functional activity of transitional B cells based on IL-10 production and CpG stimulation [40]. Given the defect in terminal stages of B-cells in SIgAD, it seems that an increase in transitional B cells and naïve B cells of our patients is due to a compensatory mechanism that augment early B cell development. Regarding different results between our study and others, it seems that this difference is due to different selection processes, as all of our patients were symptomatic, while others studied heterogeneously asymptomatic and symptomatic SIgAD patients.

Regarding T cell subsets, we observed decreased total CD4^+^ T cells, Th1, Th2, Treg cells and increased T_EMRA_ in both CD4^+^ and CD8^+^ cells. Consistent with our results, previous studies have shown an increase and reduction in CD8^+^ and CD4^+^ T lymphocytes population, respectively [4]. Also, we found that central memory in both CD4^+^ and CD8^+^ T cells and effector memory CD8^+^ T lymphocytes were decreased in SIgAD patients compared to HCs. We observed a significant increase in the T_EMRA_ cell subset in both CD4^+^ and CD8^+^ lymphocytes population, especially in severe SIgAD patients. T_EMRA_ is a third T cell memory subset in peripheral inflammatory tissues that express CD45RA but lack expression of CCR7 or CD27. In humans, T_EMRA_ cells accumulation is affected by chronic infections, such as CMV [41, 42]. An increase in these terminated T cell subsets might be due to chronic cellular response to infections in these patients; however further studies need to be performed regarding this phenomenon. Consistent with our results, Nechvatalova et al. demonstrated expanded CD4^+^ and CD8^+^ T_EMRA_ cells in SIgAD patients that were related to CMV infection [43]. We did not examine CMV infection in SIgAD patients, but an increase in the number of TEMRA cells subset in our patients could be related to chronic infections.

We recently evaluated specific antibody responses to PPSV-23 in patients with SIgAD and AT and revealed that 18.6% of the SIgAD patients and 81.3% of AT patients had an inadequate response. The number of plasmablasts, marginal zone B cells, transitional B cells, naïve CD8^+^ T cells, and percentage of CD8^+^ T cells, IgM memory B cells and switched memory B cells in SIgAD patients were significantly lower in non-responder group than responder group. Although specific antibody deficiency is more frequent in AT patients than SIgAD patients [44].

Regulatory T cells play an important role in the production of IgA antibodies by transforming growth factor-beta (TGF-β) secretion [45–47]. We found significantly decreased Tregs in our patients consistent with previous studies published [48], although one study reported increased Tregs in SIgAD patients [43]. It has also been reported a correlation between reduced Treg cells and the severity of SIgAD disease, especially in individuals with autoimmunity, and IgA CSR deficiency in patients with severe clinical manifestations [30, 48]. The low frequency of Treg cells and other T cell subsets, including Th1 and Th2 in our patients, may be due to low thymic emigrants caused by defective thymopoiesis and or increased apoptosis of these cells [49].

T cell functional assay by mitogenic or antigenic stimulation is an important feature in the diagnosis of various immune disorders and immunodeficiencies [50]. Traditionally, there is one protocol for evaluating the function of T cells based on uptake of [3H] thymidine following PHA stimulation using radioactive components that needs specific laboratory conditions and also it is not T cell-specific as it can stimulate several other immune cells as well. On the other hand, the most important weakness is that no information about specific cell subsets could be obtained. CFSE proliferation assay is a practical choice for evaluating T cell responses to an antigen or mitogen in IEI patients, especially SIgAD for targeting further potential T cell defects analyses in these patients [51]. So far, there are few reports of T-cell response defects in SIgAD patients. As expected, our study does not reveal any significant difference in T cell response between patients and controls. However, when we categorized patients into two groups based on severe and mild phenotypes, severe patients indicated decreased T cell proliferation compared to mild patients. This result could be an important finding for categorizing SIgAD patients for knowing prognosis of the patient. We recently reported that T cell proliferation was markedly impaired compared to the healthy controls in CVID patients and AT patients [29, 52]. Moreover, this indicates that SIgAD patients with defective T cell proliferation should be followed further for precise medical management. We recommend further studies for evaluation of T cell function for SIgAD patients based on severe and mild phenotypes in other studies. Limitations of the experiment included the small number of symptomatic patients, the unavailability of many of them and even the improvement of some patients.

## Conclusions

Our results indicated significant abnormalities in B cell patterns similar to CVID patients. Given that CVID and severe forms of SIgAD share almost similar clinical and immunological phenotypes and most likely genetic background, this notion is predictable. Based on phenotype analyses, we observed some more abnormalities in SIgAD patients with severe phenotypes such as a high subpopulation of CD21^low^ B cells and T cell proliferation defect. Accordingly, severe patients manifest a higher number of respiratory infections compared to mild SIgAD, with numerous numbers of those suffering from sinusitis, otitis, pneumonia and bronchiectasis, suggesting further follow-up and more precise management in these patients. The findings of the present study suggest that the investigation of B and T cell subsets could be helpful for a better understanding of the pathogenesis and prognosis of the disease.

## Supplementary Information

**Additional file 1: Table S1.** Panels of Antibodies Used for Staining of patients with SIgAD. **Table S2.** Flowcytometry results of B cell subsets in 30 SIgAD Patients. **Table S3.** Flowcytometry results of T cell subsets in 30 SIgAD Patients. **Table S4.** Correlation analysis of serum Ig levels with T cell subset in 30 patients with SIgAD. **Table S5.** Correlation analysis of absolute count and % of lymphocyte subsets in 30 patients with SIgAD.

## Data Availability

All data generated or analyzed during this study are included in this published article [and its additional files].

## References

[1] Aghamohammadi A, Mohammadi J, Parvaneh N, Rezaei N, Moin M, Espanol T (2008). Progression of selective IgA deficiency to common variable immunodeficiency. Int Arch Allergy Immunol.

[2] Bagheri Y, Sanaei R, Yazdani R, Shekarabi M, Falak R, Mohammadi J (2019). The heterogeneous pathogenesis of selective immunoglobulin a deficiency. Int Arch Allergy Immunol.

[3] Nechvatalova J, Pikulova Z, Stikarovska D, Pesak S, Vlkova M, Litzman J (2012). B-lymphocyte subpopulations in patients with selective IgA deficiency. J Clin Immunol.

[4] Litzman J, Vlková M, Pikulová Z, Štikarovská D, Lokaj J (2007). T and B lymphocyte subpopulations and activation/differentiation markers in patients with selective IgA deficiency. Clin Exp Immunol.

[5] Lemarquis AL, Einarsdottir HK, Kristjansdottir RN, Jonsdottir I, Ludviksson BR (2018). Transitional B cells and TLR9 responses are defective in selective IgA deficiency. Front Immunol.

[6] Celiksoy M, Yildiran A (2016). A comparison of B cell subsets in primary immune deficiencies that progress with antibody deficiency and age-matched healthy children. Allergol Immunopathol.

[7] Marasco E, Farroni C, Cascioli S, Marcellini V, Scarsella M, Giorda E (2017). B-cell activation with CD40L or CpG measures the function of B-cell subsets and identifies specific defects in immunodeficient patients. Eur J Immunol.

[8] Lemarquis AL, Theodors F, Einarsdottir HK, Ludviksson BR (2019). Mapping of signaling pathways linked to sIgAD reveals impaired IL-21 driven STAT3 B-cell activation. Front Immunol.

[9] Borte S, Pan-Hammarstrom Q, Liu C, Sack U, Borte M, Wagner U (2009). Interleukin-21 restores immunoglobulin production ex vivo in patients with common variable immunodeficiency and selective IgA deficiency. Blood.

[10] Abolhassani H, Kiaee F, Tavakol M, Chavoshzadeh Z, Mahdaviani SA, Momen T (2018). Fourth update on the Iranian National Registry of primary immunodeficiencies: integration of molecular diagnosis. J Clin Immunol.

[11] Aghamohammadi A, Rezaei N, Yazdani R, Delavari S, Kutukculer N, Topyildiz E (2021). Consensus Middle East and North Africa registry on inborn errors of immunity. J Clin Immunol.

[12] Seidel MG, Kindle G, Gathmann B, Quinti I, Buckland M, van Montfrans J (2019). The European Society for Immunodeficiencies (ESID) registry working definitions for the clinical diagnosis of inborn errors of immunity. J Allergy Clin Immunol Pract.

[13] Shad TM, Yousefi B, Amirifar P, Delavari S, Rae W, Kokhaei P, et al. Variable abnormalities in T and B cell subsets in ataxia telangiectasia. J Clin Immunol. 2020:1–13. 10.1007/s10875-020-00881-9 33052516

[14] TofighiZavareh F, Mirshafiey A, Yazdani R, Keshtkar AA, Abolhassani H, Bagheri Y (2021). Lymphocytes subsets in correlation with clinical profile in CVID patients without monogenic defects. Expert Rev Clin Immunol.

[15] Moeini Shad T, Yousefi B, Amirifar P, Delavari S, Rae W, Kokhaei P (2021). Variable abnormalities in T and B cell subsets in ataxia telangiectasia. J Clin Immunol.

[16] Azizi G, Mirshafiey A, Abolhassani H, Yazdani R, Ghanavatinejad A, Noorbakhsh F (2018). The imbalance of circulating T helper subsets and regulatory T cells in patients with LRBA deficiency: Correlation with disease severity. J Cell Physiol.

[17] Azizi G, Mirshafiey A, Abolhassani H, Yazdani R, Jafarnezhad-Ansariha F, Shaghaghi M (2018). Circulating helper T-cell subsets and regulatory T cells in patients with common variable immunodeficiency without known monogenic disease. J Investig Allergol Clin Immunol.

[18] Reisi M, Azizi G, Kiaee F, Masiha F, Shirzadi R, Momen T (2017). Evaluation of pulmonary complications in patients with primary immunodeficiency disorders. Eur Ann Allergy Clin Immunol.

[19] Cerutti A, Chen K, Chorny A (2011). Immunoglobulin responses at the mucosal interface. Annu Rev Immunol.

[20] Bagheri Y, Babaha F, Falak R, Yazdani R, Azizi G, Sadri M (2019). IL-10 induces TGF-β secretion, TGF-β receptor II upregulation, and IgA secretion in B cells. Eur Cytokine Netw.

[21] Ozkan H, Atlihan F, Genel F, Targan S, Gunvar T (2005). IgA and/or IgG subclass deficiency in children with recurrent respiratory infections and its relationship with chronic pulmonary damage. J Investig Allergol Clin Immunol.

[22] Tavakol M, Jamee M, Azizi G, Sadri H, Bagheri Y, Zaki-Dizaji M (2020). Diagnostic approach to the patients with suspected primary immunodeficiency. Endocr Metab Immune Disord Drug Targets.

[23] Resnick ES, Moshier EL, Godbold JH, Cunningham-Rundles C (2012). Morbidity and mortality in common variable immune deficiency over 4 decades. Blood.

[24] Yazdani R, Abolhassani H, Asgardoon M, Shaghaghi M, Modaresi M, Azizi G (2017). Infectious and noninfectious pulmonary complications in patients with primary immunodeficiency disorders. J Investig Allergol Clin Immunol.

[25] Ahmadi M, Nouri M, Babaloo Z, Farzadi L, Ghasemzadeh A, Hamdi K (2017). Intravenous immunoglobulin (IVIG) treatment modulates peripheral blood Th17 and regulatory T cells in recurrent miscarriage patients: non randomized, open-label clinical trial. Immunol Lett.

[26] Yazdani R, Seify R, Ganjalikhani-Hakemi M, Abolhassani H, Eskandari N, Golsaz-Shirazi F (2017). Comparison of various classifications for patients with common variable immunodeficiency (CVID) using measurement of B-cell subsets. Allergol Immunopathol (Madr).

[27] Aghamohammadi A, Abolhassani H, Rezaei N (2021). Primary immunodeficiency diseases in Iran: past, present and future. Arch Iran Med.

[28] Aghamohammadi A, Abolhassani H, Biglari M, Abolmaali S, Moazzami K, Tabatabaeiyan M (2011). Analysis of switched memory B cells in patients with IgA deficiency. Int Arch Allergy Immunol.

[29] Yousefi B, Amirifar P, Delavari S, Rae W, Kokhaei P, Abolhassani H (2020). Variable abnormalities in T and B cell subsets in ataxia telangiectasia. J Clin Immunol.

[30] Abolhassani H, Gharib B, Shahinpour S, Masoom SN, Havaei A, Mirminachi B (2015). Autoimmunity in patients with selective iga deficiency. J Investig Allergol Clin Immunol.

[31] Arkwright PD, Abinun M, Cant AJ (2002). Autoimmunity in human primary immunodeficiency diseases. Blood.

[32] Cerutti A, Cols M, Puga I (2013). Marginal zone B cells: virtues of innate-like antibody-producing lymphocytes. Nat Rev Immunol.

[33] Bukowska-Straková K, Kowalczyk D, Baran J, Siedlar M, Kobylarz K, Zembala M (2009). The B-cell compartment in the peripheral blood of children with different types of primary humoral immunodeficiency. Pediatr Res.

[34] Karaman SBES, Gülez N, Genel F (2018). The significance of B-cell subsets in patients with unclassified hypogammaglobulinemia and association with intravenous immunoglobulin replacement requirement. Iran J Immunol.

[35] Patuzzo G, Mazzi F, Vella A, Ortolani R, Barbieri A, Tinazzi E (2013). Immunophenotypic analysis of B lymphocytes in patients with common variable immunodeficiency: identification of CD23 as a useful marker in the definition of the disease. ISRN Immunol..

[36] Patuzzo G, Barbieri A, Tinazzi E, Veneri D, Argentino G, Moretta F (2016). Autoimmunity and infection in common variable immunodeficiency (CVID). Autoimmun Rev.

[37] Rakhmanov M, Keller B, Gutenberger S, Foerster C, Hoenig M, Driessen G (2009). Circulating CD21low B cells in common variable immunodeficiency resemble tissue homing, innate-like B cells. Proc Natl Acad Sci.

[38] Isnardi I, Ng Y-S, Menard L, Meyers G, Saadoun D, Srdanovic I (2010). Complement receptor 2/CD21−human naive B cells contain mostly autoreactive unresponsive clones. Blood.

[39] Sims GP, Ettinger R, Shirota Y, Yarboro CH, Illei GG, Lipsky PE (2005). Identification and characterization of circulating human transitional B cells. Blood.

[40] Lemarquis AL, Einarsdottir HK, Kristjansdottir RN, Jonsdottir I, Ludviksson BR (2018). Transitional B Cells and TLR9 responses are defective in selective IgA deficiency. Front Immunol.

[41] Willinger T, Freeman T, Hasegawa H, McMichael AJ, Callan MF (2005). Molecular signatures distinguish human central memory from effector memory CD8 T cell subsets. J Immunol.

[42] Martin MD, Badovinac VP (2018). Defining memory CD8 T cell. Front Immunol.

[43] Nechvatalova J, Pavlik T, Litzman J, Vlkova M (2017). Terminally differentiated memory T cells are increased in patients with common variable immunodeficiency and selective IgA deficiency. Cent Eur J Immunol.

[44] Khanmohammadi S, Shad TM, Delavari S, Shirmast P, Bagheri Y, Azizi G (2022). Evaluation of specific antibody responses in patients with selective IgA deficiency and ataxia telangiectasia. Endocr Metab Immune Disord Drug Targets.

[45] Cazac BB, Roes J (2000). TGF-β receptor controls B cell responsiveness and induction of IgA in vivo. Immunity.

[46] Van Vlasselaer P, Punnonen J, De Vries J (1992). Transforming growth factor-beta directs IgA switching in human B cells. J Immunol.

[47] Cerutti A, Rescigno M (2008). The biology of intestinal immunoglobulin A responses. Immunity.

[48] Soheili H, Abolhassani H, Arandi N, Khazaei HA, Shahinpour S, Hirbod-Mobarakeh A (2013). Evaluation of natural regulatory T cells in subjects with selective IgA deficiency: from senior idea to novel opportunities. Int Arch Allergy Immunol.

[49] Yazdani R, Fatholahi M, Ganjalikhani-Hakemi M, Abolhassani H, Azizi G, Hamid KM (2016). Role of apoptosis in common variable immunodeficiency and selective immunoglobulin A deficiency. Mol Immunol.

[50] McCusker C, Warrington R (2011). Primary immunodeficiency. All Asth Clin Immun.

[51] Marits P, Wikström A-C, Popadic D, Winqvist O, Thunberg S (2014). Evaluation of T and B lymphocyte function in clinical practice using a flow cytometry based proliferation assay. Clin Immunol.

[52] TofighiZavareh F, Mirshafiey A, Yazdani R, Keshtkar AA, Abolhassani H, Mahdaviani SA (2022). Immunophenotypic and functional analysis of lymphocyte subsets in common variable immunodeficiency patients without monogenic defects. Scand J Immunol..

