# Supplementary material for: B cells and T cells abnormalities in patients with selective IgA deficiency
Source: Allergy Asthma Clin Immunol. 2023 Mar 20;19:23. doi: 10.1186/s13223-023-00775-6 (PMC10029301; doi:10.1186/s13223-023-00775-6)
Supplement: Supplementary file 1 — Additional file 1: Table S1. Panels of Antibodies Used for Staining of patients with SIgAD. Table S2. Flowcytometry results of B cell subsets in 30 SIgAD Patients. Table S3. Flowcytometry results of T cell subsets in 30 SIgAD Patients. Table S4. Correlation analysis of serum Ig levels with T cell subset in 30 patients with SIgAD. Table S5. Correlation analysis of absolute count and % of lymphocyte subsets in 30 patients with SIgAD. [file 13223_2023_775_MOESM1_ESM.docx]

**Additional file 1**

**Table S1.** Panels of Antibodies Used for Staining of patients with SIgAD.

| **Antigen** | **Fluorochrome** | **Clone** | **Company** |
| --- | --- | --- | --- |
| **Panel (B1)** | | | |
| CD19 | APC | SJ25C1 | eBioscience |
| IgM | PerCP-eFluor™ 710 | SA-DA4 | eBioscience |
| CD27 | FITC | O323 | eBioscience |
| IgD | PE | IA6-2 | eBioscience |
| **Panel (B2)** | | | |
| CD19 | APC | SJ25C1 | eBioscience |
| IgM | PerCP-eFluor™ 710 | SA-DA4 | eBioscience |
| CD38 | FITC | HIT2 | eBioscience |
| CD21 | PE | HB5 | eBioscience |
| **Panel (T1)** | | | |
| CD4 | PerCP-Cyanine5.5 | RPA-T4 | eBioscience |
| CD8a | PerCP-Cyanine5.5 | RPA-T8 | eBioscience |
| CD45RA | FITC | JS-83 | eBioscience |
| CD197 | PE | 3D12 | eBioscience |
| **Panel (T2)** | | | |
| CD4 | PerCP-Cyanine5.5 | RPA-T4 | eBioscience |
| CD25 | APC | BC96 | eBioscience |
| CD127 | FITC | eBioRDR5 | eBioscience |
| FOXP3 | PE | 236A/E7 | eBioscience |

**Table S2:** Flowcytometry results of B cell subsets in 30 SIgAD Patients.

| **Major Clinical Manifestations** | **Plasmablast ^c^** | **CD21 low ^c^** | **Tr B ^c^** | **IgM only memory ^c^** | **SMB ^c^** | **MZB ^c^** | **B Naïve ^c^** | **BCD19**  **^b^** | **Lymph ^a^** | **Phenotype** | **Age** | **Sex** | **Patient** |  |
| --- | --- | --- | --- | --- | --- | --- | --- | --- | --- | --- | --- | --- | --- | --- |
| Pneumonia  URI | 0.5 | 1.5 | 2 | 0.6 | 3.5 | 1.4 | 80 | 11.4 | 79 | Mild | 14 | M | **P1** |  |
| Pneumonia  Rash  Allergy | 2.3 | 3.3 | 13 | 3.6 | 2.5 | 2 | 76.5 | 11.9 | 70 | Mild | 5 | M | **P2** |  |
| Pneumonia  Recurrent infection  Allergy | 1.8 | 2.5 | 30 | 7.7 | 2.5 | 2.2 | 68.5 | 13.7 | 89 | Sever | 6 | M | **P3** |  |
| Allergy | 0.9 | 4.5 | 0.5 | 1 | 13.3 | 2 | 49.7 | 13 | 43 | Mild | 18 | M | **P4** |  |
| Recurrent infection  Allergy  Tonsillectomy | 0.26 | 6.8 | 3.2 | 0 | 2.3 | 3 | 71.5 | 18.7 | 43 | Sever | 39 | M | **P5** |  |
| Allergy  FTT | 2 | 3 | 20 | 5.5 | 7 | 5 | 62 | 15.7 | 76 | Mild | 9 | M | **P6** |  |
| Sinusitis  Aphthous stomatitis  Recurrent cold | 1.7 | 3.5 | 12.5 | 6.4 | 12.2 | 4.3 | 53.2 | 12.8 | 85 | Mild | 8 | F | **P7** |  |
| Otitis  Sinusitis  Autoimmunity  Allergy | 0.7 | 0.7 | 9.2 | 4 | 1.6 | 2.3 | 83.6 | 11 | 58 | Sever | 16 | M | **P8** |  |
| Pneumonia  Recurrent infection  Allergy | 0.9 | 1.5 | 14.1 | 1.3 | 3 | 2.5 | 73 | 9.4 | 30 | Sever | 6 | M | **P9** |  |
| Recurrent Sinusitis  Pneumonia  Aphthous stomatitis  Allergy  Recurrent infection | 0.7 | 1.2 | 7 | 0.7 | 0.38 | 2 | 87.2 | 11 | 21 | Sever | 12 | M | **P10** |  |
| Thyroid abnormality Eye, nail and skin infection | 1.5 | 6 | 1.7 | 3 | 4.83 | 1 | 70.5 | 9.11 | 31 | Sever | 46 | F | **P11** |  |
| Cold  Cough | 1.3 | 6 | 8 | 6 | 4.11 | 2.5 | 62.8 | 7.34 | 40 | Mild | 7 | M | **P12** |  |
| Asymptomatic | 0.2 | 1.5 | 3.9 | 2 | 1.9 | 3 | 80 | 15.7 | 71 | Mild | 9 | M | **P13** |  |
| Pneumonia, Eyes infection, Otitis media, Diarrhea | 0.3 | 23.6 | 0.8 | 0.36 | 6 | 20 | 29.5 | 10.6 | 41 | Sever | 38.7 | M | **P14** |  |
| Recurrent Sinusitis  Gastrointestinal disorder | 0.5 | 4 | 0.3 | 4 | 2 | 2 | 77 | 14.6 | 39 | Sever | 7.5 | M | **P15** |  |
| Vitiligo  Asthma  Sinusitis | 0.9 | 3 | 9.6 | 10 | 3.8 | 3 | 64.2 | 11.7 | 70 | Sever | 10 | F | **P16** |  |
| Diarrhea  Allergy  UTI | 0.6 | 1 | 3.4 | 0.6 | 2 | 1.2 | 83.4 | 11.9 | 74 | Mild | 12 | F | **P17** |  |
| Epilepsy  Asthma  Pneumonia  Allergy, Otitis media, Sinusitis | 0.2 | 2 | 6.6 | 0.7 | 1.7 | 1.5 | 84.3 | 9.56 | 66 | Sever | 22 | F | **P18** |  |
| Diarrhea | 0.5 | 0.3 | 15.5 | 2.5 | 0.91 | 1 | 86.3 | 9.4 | 69 | Mild | 10 | M | **P19** |  |
| Asthma  Allergy |  |  |  |  |  |  | 66 | 8.5 | 87 | Mild | 27 | M | **P20** |  |
| Cough | 9 | 1.6 | 6 | 3.5 | 1.71 | 2.5 | 68.3 | 10 | 78 | Mild | 15 | M | **P21** |  |
| Allergy  Diarrhea | 1.7 | 2.3 | 33.4 | 2.5 | 3.7 | 2 | 72.9 | 12.4 | 67 | Mild | 4.8 | F | **P22** |  |
| Asthma  Allergy  Diarrhea | 0.5 | 0.6 | 11.1 | 0.7 | 1.44 | 1.6 | 82 | 11.4 | 81 | Mild | 12 | M | **P23** |  |
| Diarrhea | 1 | 6 | 10.7 | 1.5 | 15 | 16 | 44 | 8.84 | 76 | Mild | 10 | M | **P24** |  |
| Pneumonia  Diarrhea | 1 | 2 | 8 | 1.5 | 6 | 2 | 73.9 | 4.34 | 77 | Mild | 14 | M | **P25** |  |
| Asthma  Allergy  Pneumonia | 0.4 | 4.5 | 25.2 | 1.2 | 4.4 | 4 | 68.8 | 9.9 | 70 | Sever | 16 | M | **P26** |  |
| Eyes infection  Abscess | 1.2 | 2.1 | 7.6 | 4.4 | 5 | 2.2 | 50.3 | 10.1 | 45 | Sever | 5 | M | **P27** |  |
| Allergy  Pneumonia | 0.4 | 6.4 | 4 | 0.8 | 8.2 | 4.4 | 73 | 14.3 | 86 | Mild | 9 | M | **P28** |  |
| Pneumonia  Diarrhea | 0.7 | 1.6 | 11.1 | 4.2 | 3.3 | 3 | 65 | 13.3 | 55 | Mild | 5 | F | **P29** |  |
| Allergy | 0.5 | 1 | 14.7 | 1.5 | 3.6 | 7 | 64 | 7 | 53 | Mild | 16 | M | **P30** |  |
| *^a^:% of total peripheral blood mononuclear cells, ^b^: % of Lymphocyte population, ^c^: % of CD8+ T cells,  ^d^: % of CD4+ T cells, ^e^: % of CD19+ B cells*  *White box: Normal, Red box: Higher than normal range, Blue box: Lower than normal range* | | | | | | | | | | | | | | |

**Table S3:** Flowcytometry results of T cell subsets in 30 SIgAD Patients.

| **Major Clinical Manifestations** | **Th17** | **Th2** | **Th1** | **Treg ^d^**  **(CD127^-/low^)** | **T4 Effector memory ^d^** | **T4 TEMRA ^d^** | **T4 Central Memory ^d^** | **T4 Naïve ^d^** | **T4 ^b^** | **T8 Effector memory ^c^** | **T8 TEMRA ^c^** | **T8 Central Memory ^c^** | **T8 Naïve ^c^** | **T8 ^b^** | **Lymph ^a^** | **Phenotype** | **Age** | **Sex** | **Patient** |  |
| --- | --- | --- | --- | --- | --- | --- | --- | --- | --- | --- | --- | --- | --- | --- | --- | --- | --- | --- | --- | --- |
| Pneumonia  URI  sore throat | 1.18 | 0.49 | 6.8 | 1.2 | 5.8 | 1.5 | 12.7 | 80 | 51.6 | 7.82 | 19.2 | 1.25 | 71.8 | 24.2 | 79 | Mild | 14 | M | **P1** |  |
| Pneumonia  Rash  Allergy | 1.15 | 0.49 | 7.65 | 0.3 | 5.8 | 17.2 | 11.4 | 65.7 | 31.7 | 9.16 | 57.9 | 0.29 | 32.7 | 30.1 | 70 | Mild | 5 | M | **P2** |  |
| Pneumonia  Recurrent infection  Allergy | 0.84 | 0.47 | 6.93 | 1.1 | 5.6 | 8.6 | 9.8 | 76 | 35.9 | 4.87 | 48.2 | 0.47 | 46.4 | 23.5 | 89 | Sever | 6 | M | **P3** |  |
| Allergy | 0.88 | 0.23 | 8.77 | 0 | 46.6 | 9 | 13.2 | 31.2 | 31 | 23.1 | 44 | 0.3 | 32.6 | 31.5 | 43 | Mild | 18 | M | **P4** |  |
| Recurrent infection  Allergy  Tonsillectomy | 1.81 | 0.55 | 8.19 | 1.6 | 25 | 3.5 | 37.8 | 33.7 | 19.6 | 41.8 | 28.3 | 7.74 | 22.1 | 13.8 | 43 | Sever | 39 | M | **P5** |  |
| Allergy  FTT | 1 | 0.19 | 7.64 | 0 | 30.6 | 17 | 10.2 | 42.1 | 25.4 | 32 | 32 | 1.07 | 98.9 | 21.9 | 76 | Mild | 9 | M | **P6** |  |
| Sinusitis  Aphthous stomatitis  Recurrent cold | 1.17 | 0.23 | 7.89 | 0 | 5.8 | 3.1 | 12.7 | 78.4 | 30.2 | 26.7 | 28.5 | 0.48 | 44.3 | 26.8 | 85 | Mild | 8 | F | **P7** |  |
| Otitis  Sinusitis  Autoimmunity  Allergy | 1 | 0.37 | 12.6 | 0.9 | 5.7 | 5.5 | 17.5 | 71.4 | 41.9 | 12.1 | 38.9 | 0.83 | 48.1 | 20.8 | 58 | Sever | 16 | M | **P8** |  |
| Pneumonia  Recurrent infection  Allergy | 0.49 | 1 | 2.43 | 0.2 | 8.8 | 3.3 | 9.2 | 78.8 | 28.9 | 6.28 | 23.9 | 0.71 | 69.1 | 17.1 | 30 | Sever | 6 | M | **P9** |  |
| Recurrent Sinusitis  Pneumonia  Aphthous stomatitis  Allergy  Recurrent infection | 2 | 0.24 | 4.31 | 0.1 | 8.3 | 4.1 | 10.7 | 77 | 27.3 | 3.08 | 38.9 | 0.67 | 57.3 | 17.2 | 21 | Sever | 12 | M | **P10** |  |
| thyroid abnormality Eye, nail and skin infection | 1.37 | 0.41 | 15 | 0.7 | 52.8 | 16.3 | 11 | 19.9 | 36.4 | 16 | 55.6 | 0.67 | 27.8 | 16.1 | 31 | Sever | 46 | F | **P11** |  |
| Cold  Cough | 0.86 | 0.45 | 5.85 | 0.4 | 13.5 | 9 | 6.9 | 70.7 | 38.9 | 7.79 | 37.4 | 0.46 | 54.3 | 27.8 | 40 | Mild | 7 | M | **P12** |  |
| Asymptomatic | 0.75 | 0.38 | 5.64 | 1.1 | 22.5 | 29 | 6.2 | 42.3 | 31.5 | 7.51 | 45 | 0.14 | 47.3 | 30.9 | 71 | Mild | 9 | M | **P13** |  |
| Pneumonia, Eyes infection, Otitis media, Diarrhea | 2 | 0.11 | 26.8 | 0.63 | 32.8 | 10.1 | 38.3 | 18.8 | 48.8 | 22.1 | 56 | 3.16 | 18.7 | 30.6 | 41 | Sever | 38.7 | M | **P14** |  |
| Recurrent Sinusitis  Gastrointestinal disorder | 1.46 | 0.25 | 5 | 0 | 22.8 | 12.3 | 12.5 | 52.3 | 38.4 | 11.1 | 69.8 | 0.56 | 18.6 | 28.6 | 39 | Sever | 7.5 | M | **P15** |  |
| Vitiligo  Asthma  Sinusitis | 1.67 | 0.24 | 3.19 | 0 | 20.2 | 20.5 | 6.2 | 53.1 | 28.2 | 22.4 | 44.3 | 0.22 | 33 | 29 | 70 | Sever | 10 | F | **P16** |  |
| Diarrhea  Allergy  UTI | 1.3 | 0.73 | 5 | 0.64 | 15 | 11.3 | 11.6 | 62.1 | 39.4 | 6.98 | 22.5 | 0.55 | 69.9 | 33.7 | 74 | Mild | 12 | F | **P17** |  |
| Epilepsy  Asthma  Pneumonia  Allergy, Otitis media, Sinusitis | 0.91 | 0.58 | 12.6 | 0.8 | 24.2 | 23.8 | 7.4 | 44.6 | 44.8 | 12.6 | 66.2 | 0.44 | 20.8 | 30.9 | 66 | Sever | 22 | F | **P18** |  |
| Diarrhea | 1.13 | 0.19 | 9.65 | 0 | 16.8 | 6.2 | 13.1 | 63.9 | 27.4 | 7.51 | 46.3 | 0.41 | 45.8 | 36 | 69 | Mild | 10 | M | **P19** |  |
| Asthma  Allergy | 10 | 1 | 15 | 0.1 | 75 | 18 | 4 | 32 | 38 | 31 | 61 | 0.7 | 7 | 25 | 87 | Mild | 27 | M | **P20** |  |
| Cough | 1 | 0.39 | 6.93 | 0.2 | 11.7 | 5.9 | 11.1 | 71.3 | 52.2 | 9.03 | 42.5 | 0.88 | 47.6 | 23.1 | 78 | Mild | 15 | M | **P21** |  |
| Allergy  Diarrhea | 1 | 0.38 | 8.88 | 0.2 | 15.4 | 13.5 | 6 | 65.1 | 53.6 | 16.4 | 45.7 | 0.3 | 37.6 | 18.3 | 67 | Mild | 4.8 | F | **P22** |  |
| Asthma  Allergy  Diarrhea | 3.25 | 0.24 | 8.8 | 0.4 | 32.2 | 7.1 | 19.7 | 41 | 39.1 | 19.6 | 39.8 | 1.42 | 39.2 | 19.4 | 81 | Mild | 12 | M | **P23** |  |
| Diarrhea | 3.19 | 0.3 | 4 | 0.5 | 30.3 | 29.3 | 6.3 | 34.1 | 36.6 | 25.5 | 57.1 | 0.17 | 17.2 | 43 | 76 | Mild | 10 | M | **P24** |  |
| Pneumonia  Diarrhea | 1.41 | 0.28 | 8.26 | 0 | 29.3 | 11.1 | 11.1 | 48.5 | 35.6 | 19.5 | 49.1 | 0.34 | 31.1 | 34.6 | 77 | Mild | 14 | M | **P25** |  |
| Asthma  Allergy  Pneumonia | 1.12 | 0.35 | 13.2 | 0.3 | 16.2 | 6.2 | 11 | 66.2 | 32.3 | 13.2 | 35.2 | 0.45 | 22.3 | 30.2 | 70 | Sever | 16 | M | **P26** |  |
| Eyes infection  Abscess | 0.85 | 0.41 | 5.3 | 1.6 | 24.3 | 10.2 | 10.4 | 73.1 | 40.4 | 7.7 | 57.2 | 0.72 | 45.6 | 21.5 | 45 | Sever | 5 | M | **P27** |  |
| Allergy  Pneumonia | 1.5 | 0.66 | 10.7 | 0 | 14.2 | 9 | 16.3 | 35.3 | 50.2 | 22.6 | 25.5 | 0.9 | 65.7 | 17.3 | 86 | Mild | 9 | M | **P28** |  |
| Pneumonia  Diarrhea | 1.8 | 0.22 | 9.4 | 0.5 | 6.3 | 16.2 | 7.1 | 55.2 | 35.8 | 15.2 | 44.2 | 0.66 | 44.2 | 18.6 | 55 | Mild | 5 | F | **P29** |  |
| Allergy | 1.9 | 0.26 | 4.7 | 0.2 | 16 | 1.5 | 14 | 59 | 41 | 7.9 | 20 | 0.8 | 71 | 31 | 53 | Mild | 16 | M | **P30** |  |
| *^a^:% of total peripheral blood mononuclear cells, ^b^: % of Lymphocyte population, ^c^: % of CD8+ T cells,  ^d^: % of CD4+ T cells, ^e^: % of CD19+ B cells*  *White box: Normal, Red box: Higher than normal range, Blue box: Lower than normal range* | | | | | | | | | | | | | | | | | | | | |

| **Table S4:** Correlation analysis of serum Ig levels with T cell subset in 30 patients with SIgAD. | | | | | |
| --- | --- | --- | --- | --- | --- |
| **T cell subsets** |  | **IgM** | **IgG** | **IgA** | **IgE** |
| TCD8 | Pearson's r | -0.27 | -0.19 | -0.18 | -0.21 |
|  | p-value | 0.2035 | 0.362232 | 0.37575 | 0.34899 |
| TCD8_Naïve | Pearson's r | 0.23 | -0.23 | 0.15 | 0.33 |
|  | p-value | 0.2737 | 0.022254* | 0.46042 | 0.13234 |
| TCD8_CM | Pearson's r | -0.2 | 0.17 | -0.25 | -0.1 |
|  | p-value | 0.3559 | 0.431557 | 0.21594 | 0.66071 |
| TCD8_TEMRA | Pearson's r | -0.07 | 0.11 | -0.07 | -0.26 |
|  | p-value | 0.7338 | 0.599395 | 0.72576 | 0.24135 |
| TCD8_EM | Pearson's r | -0.3 | 0.2 | -0.27 | -0.26 |
|  | p-value | 0.1495 | 0.337038 | 0.16624 | 0.2495 |
| TCD4 | Pearson's r | -0.12 | -0.06 | -0.2 | -0.22 |
|  | p-value | 0.5733 | 0.781019 | 0.32758 | 0.32013 |
| TCD4_Naïve | Pearson's r | -0.15 | -0.35 | 0.3 | 0.27 |
|  | p-value | 0.4962 | 0.012487* | 0.1234 | 0.22655 |
| TCD4_CM | Pearson's r | -0.28 | 0.02 | -0.24 | -0.17 |
|  | p-value | 0.1902 | 0.929402 | 0.23529 | 0.45872 |
| TCD4_TEMRA | Pearson's r | 0.24 | -0.02 | 0.11 | -0.15 |
|  | p-value | 0.2588 | 0.910102 | 0.56891 | 0.50788 |
| TCD4_EM | Pearson's r | 0.15 | 0.68 | -0.39 | -0.22 |
|  | p-value | 0.4857 | 2.90E-04** | 0.06475 | 0.31696 |
| Treg | Pearson's r | -0.03 | -0.01 | -0.16 | -0.08 |
|  | p-value | 0.8755 | 0.950592 | 0.43571 | 0.72066 |
| Th1percent | Pearson's r | -0.05 | 0.17 | -0.27 | -0.3 |
|  | p-value | 0.8292 | 0.430358 | 0.17225 | 0.1779 |
| Th2P | Pearson's r | 0.05 | 0.46 | -0.11 | 0.53 |
|  | p-value | 0.8256 | 0.024286 | 0.57861 | 0.01082 |
| Th2Q | Pearson's r | -0.18 | 0.21 | -0.15 | -0.12 |
|  | p-value | 0.4103 | 0.321208 | 0.44194 | 0.60555 |
| Th17P | Pearson's r | -0.24 | 0.63 | -0.12 | -0.21 |
|  | p-value | 0.2664 | 9.17E-04** | 0.56696 | 0.35012 |

| **Table S5:** Correlation analysis of absolute count and % of lymphocyte subsets in 30 patients with SIgAD. | | | | | | | | | | | | | | | | | | | | | | |
| --- | --- | --- | --- | --- | --- | --- | --- | --- | --- | --- | --- | --- | --- | --- | --- | --- | --- | --- | --- | --- | --- | --- |
| **Parameters** |  | **TCD8-abs** | **TCD8**  **Naive abs** | **TCD8 CM abs** | **TCD8 TEMRA abs** | **TCD8_EM_ abs** | **TCD4_ abs** | **TCD4_Naive_ abs** | **TCD4_CM_ abs** | **TCD4_TEMRA_ abs** | **TCD4_EM_ abs** | **Treg_ abs** | **Th1 abs** | **Th2 abs** | **Th17 abs** | **BCD19_ abs** | **B_Naive_ abs** | **MZB_ abs** | **SMB_ abs** | **IgM_memorye_ abs** | **TrB_ abs** | **CD21low_ abs** |
| TCD8_Naïve_% | Pearson's r | -0.18 |  |  |  |  |  |  |  |  |  |  |  |  |  |  |  |  |  |  |  |  |
|  | p-value | 0.17725 |  |  |  |  |  |  |  |  |  |  |  |  |  |  |  |  |  |  |  |  |
| TCD8_CM_% | Pearson's r | -0.42 | -0.36 |  |  |  |  |  |  |  |  |  |  |  |  |  |  |  |  |  |  |  |
|  | p-value | 7.90E-04 | 0.005299 |  |  |  |  |  |  |  |  |  |  |  |  |  |  |  |  |  |  |  |
| TCD8_TEMRA_% | Pearson's r | 0.38 | -0.12 | -0.24 |  |  |  |  |  |  |  |  |  |  |  |  |  |  |  |  |  |  |
|  | p-value | 0.003019 | 0.361703 | 0.059223 |  |  |  |  |  |  |  |  |  |  |  |  |  |  |  |  |  |  |
| TCD8_EM_% | Pearson's r | -0.24 | -0.47 | 0.34 | -0.2 |  |  |  |  |  |  |  |  |  |  |  |  |  |  |  |  |  |
|  | p-value | 0.061199 | 1.40E-04 | 0.008522 | 0.134229 |  |  |  |  |  |  |  |  |  |  |  |  |  |  |  |  |  |
| TCD4_% | Pearson's r | -0.09 | -0.03 | 0.26 | -0.09 | -0.04 |  |  |  |  |  |  |  |  |  |  |  |  |  |  |  |  |
|  | p-value | 0.477771 | 0.833221 | 0.045021 | 0.492807 | 0.748012 |  |  |  |  |  |  |  |  |  |  |  |  |  |  |  |  |
| TCD4_Naïve_% | Pearson's r | 0.17 | 0.37 | -0.28 | 0.06 | -0.24 | 0.21 |  |  |  |  |  |  |  |  |  |  |  |  |  |  |  |
|  | p-value | 0.181304 | 0.003622 | 0.029042 | 0.649054 | 0.067011 | 0.10596 |  |  |  |  |  |  |  |  |  |  |  |  |  |  |  |
| TCD4_CM_% | Pearson's r | -0.37 | -0.28 | 0.69 | -0.38 | -0.09 | -0.32 | -0.31 |  |  |  |  |  |  |  |  |  |  |  |  |  |  |
|  | p-value | 0.00412 | 0.027568 | 1.13E-09 | 0.002555 | 0.482339 | 0.01213 | 0.0145 |  |  |  |  |  |  |  |  |  |  |  |  |  |  |
| TCD4_TEMRA_% | Pearson's r | 0.37 | 0.11 | -0.49 | 0.55 | 0.19 | 0.2 | 0 | -0.08 |  |  |  |  |  |  |  |  |  |  |  |  |  |
|  | p-value | 0.00327 | 0.383397 | 6.59E-05 | 6.65E-06 | 0.139269 | 0.11773 | 0.997142 | 0.549622 |  |  |  |  |  |  |  |  |  |  |  |  |  |
| TCD4_EM_% | Pearson's r | -0.02 | -0.32 | 0.05 | 0.11 | 0.36 | -0.08 | -0.44 | -0.11 | 0.23 |  |  |  |  |  |  |  |  |  |  |  |  |
|  | p-value | 0.899653 | 0.01381 | 0.684023 | 0.388648 | 0.004224 | 0.55914 | 3.77E-04 | 0.402074 | 0.073292 |  |  |  |  |  |  |  |  |  |  |  |  |
| Treg_% | Pearson's r | -0.36 | -0.29 | 0.45 | -0.31 | -0.19 | -0.32 | -0.25 | -0.21 | -0.44 | -0.25 |  |  |  |  |  |  |  |  |  |  |  |
|  | p-value | 0.004465 | 0.02559 | 2.98E-04 | 0.01559 | 0.151175 | 0.01158 | 0.05507 | 0.107156 | 3.81E-04 | 0.056575 |  |  |  |  |  |  |  |  |  |  |  |
| Th1 % | Pearson's r | -0.26 | -0.31 | 0.16 | -0.2 | -0.03 | -0.12 | -0.21 | 0.08 | -0.17 | 0.1 | 0 |  |  |  |  |  |  |  |  |  |  |
|  | p-value | 0.045637 | 0.014955 | 0.227858 | 0.122949 | 0.817897 | 0.34813 | 0.101954 | 0.534177 | 0.194894 | 0.446373 | 0.9842 |  |  |  |  |  |  |  |  |  |  |
| Th2% | Pearson's r | -0.23 | -0.22 | 0.32 | -0.25 | 0.12 | -0.19 | -0.24 | -0.18 | -0.22 | 0.04 | 0.09 | -0.13 |  |  |  |  |  |  |  |  |  |
|  | p-value | 0.080943 | 0.094558 | 0.011319 | 0.050533 | 0.380349 | 0.14191 | 0.06593 | 0.177132 | 0.098135 | 0.733118 | 0.5039 | 0.33686 |  |  |  |  |  |  |  |  |  |
| Th17% | Pearson's r | 0.21 | -0.11 | -0.11 | 0.33 | 0.44 | 0.22 | -0.13 | 0.06 | 0.38 | 0.75 | -0.17 | 0.36 | 0.27 |  |  |  |  |  |  |  |  |
|  | p-value | 0.106227 | 0.414533 | 0.423157 | 0.011153 | 4.22E-04 | 0.09505 | 0.319657 | 0.631417 | 0.002549 | 3.64E-12 | 0.1873 | 0.004815 | 0.03554 |  |  |  |  |  |  |  |  |
| BCD19_% | Pearson's r | 0.3 | 0.35 | -0.22 | 0.24 | 0.04 | 0.3 | 0.26 | 0.29 | 0.35 | 0.04 | -0.05 | 0.09 | -0.09 | 0.06 |  |  |  |  |  |  |  |
|  | p-value | 0.019164 | 0.006206 | 0.089485 | 0.069515 | 0.741034 | 0.02151 | 0.040867 | 0.025899 | 0.006276 | 0.787031 | 0.7081 | 0.498606 | 0.51249 | 0.649098 |  |  |  |  |  |  |  |
| B_Naïve_% | Pearson's r | 0.1 | 0.16 | -0.24 | 0.09 | -0.12 | 0.17 | 0.2 | 0.06 | 0.19 | 0 | -0.03 | 0.03 | 0.09 | 0.06 | 0.2 |  |  |  |  |  |  |
|  | p-value | 0.441612 | 0.211648 | 0.067967 | 0.473402 | 0.359606 | 0.19221 | 0.126135 | 0.67043 | 0.14552 | 0.981319 | 0.7992 | 0.806362 | 0.4765 | 0.675784 | 0.128038 |  |  |  |  |  |  |
| MZB_% | Pearson's r | 0.02 | -0.14 | 0.4 | 0.02 | 0.33 | -0.07 | -0.22 | 0.12 | -0.02 | 0.19 | 0.1 | 0.1 | 0.28 | 0.07 | -0.15 | -0.25 |  |  |  |  |  |
|  | p-value | 0.884615 | 0.279177 | 0.001545 | 0.91018 | 0.010931 | 0.58386 | 0.099156 | 0.351615 | 0.857532 | 0.139859 | 0.4564 | 0.471691 | 0.02868 | 0.619678 | 0.244954 | 0.053673 |  |  |  |  |  |
| SMB_% | Pearson's r | 0.01 | -0.06 | 0.39 | -0.08 | 0.35 | -0.09 | -0.15 | -0.06 | -0.21 | 0.05 | 0.07 | 0.02 | 0.14 | -0.05 | -0.15 | -0.29 | 0.31 |  |  |  |  |
|  | p-value | 0.967984 | 0.672513 | 0.002369 | 0.531485 | 0.006978 | 0.50116 | 0.262345 | 0.62847 | 0.112323 | 0.697341 | 0.5846 | 0.859767 | 0.28746 | 0.722561 | 0.25705 | 0.028123 | 0.017079 |  |  |  |  |
| IgM_memory_% | Pearson's r | -0.07 | 0.03 | -0.14 | -0.11 | -0.08 | -0.11 | -0.01 | -0.19 | -0.06 | -0.2 | -0.02 | 0.01 | -0.05 | -0.15 | -0.04 | -0.09 | -0.11 | -0.01 |  |  |  |
|  | p-value | 0.600976 | 0.799647 | 0.300529 | 0.424751 | 0.566241 | 0.4237 | 0.966495 | 0.148503 | 0.676506 | 0.138185 | 0.8861 | 0.94639 | 0.70275 | 0.25035 | 0.735432 | 0.51573 | 0.418113 | 0.944398 |  |  |  |
| TrB_% | Pearson's r | 0.33 | 0.32 | -0.3 | 0.26 | -0.03 | 0.27 | 0.37 | 0.03 | 0.32 | -0.05 | -0.13 | 0.12 | -0.03 | 0.15 | 0.41 | 0.41 | 0.17 | 0.13 | 0.47 |  |  |
|  | p-value | 0.010351 | 0.013015 | 0.021431 | 0.045332 | 0.806357 | 0.03646 | 0.003849 | 0.826761 | 0.012846 | 0.722732 | 0.3389 | 0.357711 | 0.81431 | 0.256997 | 0.001258 | 0.001318 | 0.208768 | 0.323548 | 1.97E-04 |  |  |
| CD21lowe_% | Pearson's r | 0.02 | -0.13 | 0 | 0.1 | 0.18 | -0.01 | -0.14 | 0.29 | 0.11 | 0.19 | -0.15 | 0.3 | -0.09 | 0.05 | 0.05 | -0.03 | 0.5 | 0.14 | 0 | -0.03 |  |
|  | p-value | 0.854263 | 0.314705 | 0.987173 | 0.448161 | 0.182041 | 0.95058 | 0.287712 | 0.026468 | 0.40479 | 0.14435 | 0.2702 | 0.022816 | 0.47585 | 0.693807 | 0.700069 | 0.835928 | 6.38E-05 | 0.288797 | 0.988923 | 0.832505 |  |
| Plasmablaste % | Pearson's r | -0.02 | 0.03 | 0.01 | -0.01 | -0.08 | 0.14 | 0.23 | 0.03 | -0.07 | -0.09 | -0.03 | 0.09 | 0.01 | 0.03 | 0.02 | -0.01 | -0.04 | 0.03 | 0.1 | 0.05 | -0.11 |
|  | p-value | 0.906093 | 0.81823 | 0.957526 | 0.941491 | 0.542226 | 0.30392 | 0.083936 | 0.832948 | 0.586151 | 0.519191 | 0.8203 | 0.474812 | 0.94484 | 0.818971 | 0.895051 | 0.915258 | 0.739358 | 0.818089 | 0.434581 | 0.726607 | 0.4284 |
